# Antimicrobial use practices and resistance in indicator bacteria in communal cattle in the Mnisi community, Mpumalanga, South Africa

**DOI:** 10.1002/vms3.334

**Published:** 2020-08-31

**Authors:** Charlotte Ropafadzo Mupfunya, Daniel Nenene Qekwana, Vinny Naidoo

**Affiliations:** ^1^ Department of Veterinary Tropical Diseases University of Pretoria Onderstepoort South Africa; ^2^ Department of Paraclinical Studies University of Pretoria Onderstepoort South Africa

**Keywords:** antimicrobial resistance, antimicrobial use, communal farming, *Enterococcus*, *Escherichia coli*, surveillance

## Abstract

Surveillance of antimicrobial use and antimicrobial resistance is a critical component of the “Global Action Plan” to tackle antimicrobial resistance. However, there is a paucity of such surveillance in communal farming areas in South Africa. This study investigated knowledge and antimicrobial use practices by cattle farmers and antimicrobial resistance levels of indicator bacteria in cattle in a rural communal farming area in South Africa. Seventy (70) farmers were interviewed at five cattle inspection sites using structured questionnaires. Rectal swabs were collected from apparently healthy cattle (*n* = 100) for culture of *Escherichia coli* and *Enterococcus* species, and antimicrobial susceptibility testing using broth microdilution. The farmers indicated predominantly using tetracyclines. Although 42% of the farmers indicated hearing about antimicrobial resistance, none of them clearly understood what it involves. Seventy‐nine (79) *E. coli* and 71 *Enterococcus* species were isolated with *E. faecium* being the dominant species among the *Enterococcus* isolates. *E. coli* isolates were resistant to colistin (16%; 13/79), chlortetracycline (8%; 6/79) and amoxicillin (8%; 6/79). *Enterococcus* isolates were resistant to enrofloxacin (55%; 39/71) and amoxycillin (3%; 2/71). We observed knowledge gaps in prudent antimicrobial use practices and antimicrobial resistance among the farmers. Farmer tailored education programmes on primary animal health care and prudent antimicrobial use practices must be developed and implemented to improve antimicrobial stewardship among farmers with limited veterinary supervision. The level of colistin resistance detected among *E. coli* isolates from rural communal cattle in this study was unexpected and warrants further molecular investigation to check if the resistance is plasmid mediated.

## Graphical abstract

This study investigated knowledge and antimicrobial use practices by farmers and antimicrobial resistance levels of indicator bacteria in cattle in a communal farming community in South Africa. The level of antimicrobial resistance detected was generally low. However, imprudent antimicrobial use practices and knowledge gaps in antimicrobial resistance were identified among the farmers thereby highlighting the need for farmer‐tailored education programmes.

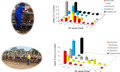

## 1 INTRODUCTION

Livestock rearing in communal farming areas in South Africa is an important means of diversifying livelihoods and alleviating poverty (Meissner, Scholtz, & Palmer, 2013). In communal farming areas, livestock are kept on separate households at night but graze together on communal pastures. The communal farming system has several challenges including high disease occurrence and poor veterinary extension services (Mutibvu, Maburutse, Mbiriri, & Kashangura, 2012). As a countermeasure, the South African government through the Fertilizers, Farm Feeds, Agricultural Remedies and Stock Remedies Act 36 of 1947 permits for over‐the‐counter availability of antimicrobials such as tetracyclines, sulphonamides, cloxacillin intramammary, fosfomycin, tylosin and kitasamycin, to allow for timely treatment of easily recognisable endemic diseases (Henton, Eagar, Swan, & van Vuuren, 2011; Naidoo, 2009).

Unfortunately, availing antimicrobials for use by farmers without much restriction increases the likelihood of indiscriminate antimicrobial use, a recognised key driver of antimicrobial resistance development (Aarestrup, 2015). Antimicrobial resistance is on the increase and is currently a critical threat to human and animal health due to the associated increased health care expenses, treatment failure and high mortalities (Roca et al., 2015).

Surveillance is one of the five strategic objectives outlined in the World Health Organisation (WHO) “Global Action Plan” for tackling antimicrobial resistance (WHO, 2015a). It allows for timely detection of emerging resistance and drivers of resistance, and guides intervention strategies (Aarestrup, 2015). *Escherichia coli* and enterococci have been selected as indicator bacteria for monitoring resistance because they easily develop resistance (Caprioli, Busani, Martel, & Helmuth, 2000; van Vuuren, Picard, & Greyling, 2007) and also serve as reservoirs of antimicrobial resistance genes which are transferrable to pathogenic bacteria (Caprioli et al., 2000; Varga et al., 2008). In addition, these microorganisms are present in both healthy and sick individuals and therefore provide a better picture of the antimicrobial resistance patterns in a population compared to pathogenic bacteria (Caprioli et al., 2000).

Livestock farmers in South Africa have access to certain antimicrobials without veterinary supervision. However, there is paucity of information on their knowledge of antimicrobials and antimicrobial use practices, more so for communal farmers. In addition, data on antimicrobial resistance patterns of indicator bacteria in communal cattle are also limited. Therefore, this study investigated knowledge on antimicrobial use practices and antimicrobial resistance levels of indicator bacteria in cattle in a communal farming community in South Africa. Information generated from this study can be used to guide policy on the availability of over the counter veterinary antimicrobials in South Africa to contribute towards prudent antimicrobial use.

## 2 MATERIALS AND METHODS

### 2.1 The study area

The study was conducted in the Mnisi community, a rural communal farming community in the Bushbuckridge Municipality area, Mpumalanga Province South Africa. The community spans over 29,500 hectares of land and is subdivided into four wards which consist of an estimated 8,500 homesteads with over 40,000 individuals. The municipality is characterised by high unemployment and low household incomes. Animal husbandry is the major agricultural activity in the area with cattle farming dominating (Berrian et al., 2016). The livestock herds share the same grazing areas (i.e. communal pastures) during the day but are penned at the owners’ households during the night. The area is semi‐arid hence most livestock are prone to water and pasture shortages. Tick‐borne diseases such as corridor disease, anaplasmosis, heartwater and babesiosis commonly occur in the area (Choopa, 2016).

Over two thirds of the community's borders are shared with game parks. Consequently, there is spill over of foot and mouth disease (FMD) and tick‐borne diseases from wildlife to livestock and vice versa. The cattle are thus required by law to be presented weekly for tick control using acaricides, and for inspection for foot and mouth disease lesions at designated communal inspection sites (Berrian et al., 2016). There were 21 inspection sites distributed across the four wards.

Each ward is serviced by one Animal health technician (AHT) (a veterinary paraprofessional) who serves as the primary veterinary personnel contact for the communal farmers. The farmers can buy veterinary drugs from the local animal clinic or an agricultural cooperative store in a neighbouring town which sells agricultural supplies including over‐the‐counter antimicrobials such as tetracyclines and sulphonamides. For technical reasons, sample collection was limited to all the five inspection sites of one ward.

### 2.2 Study population

The study was conducted from January to February 2018. Invitation to participate in the study was extended by word of mouth to farmers at the five cattle inspection sites. All farmers willing to participate were interviewed and their cattle selected for sample collection. In total, 70 farmers were included in the study.

### 2.3 Questionnaire survey

Face‐to‐face interviews based on a standardised questionnaire, translated to the local language, were conducted with participating farmers with the assistance of a local environmental health monitor (EHM). The farmers were interviewed using Shangaan, which is the local language and their responses to open‐ended questions were translated by the EHM. The translated questionnaire was pretested using a mock interview prior to the survey to ensure that the meaning of the questions was not lost during translation.

### 2.4 Farmer questionnaire

The questionnaire included both open and close‐ended questions for demographic data, livestock species kept and duration of cattle rearing, cattle herd size, antimicrobials used on the farm and where they source them, disposal of expired antimicrobials and knowledge on antimicrobial resistance and importance of observing antimicrobial withdrawal periods.

### 2.5 Animal health technician questionnaire and veterinarian interviews

A questionnaire (supporting information) with a different set of questions from the farmers’ questionnaire was administered to two AHTs working in the area to obtain information on antimicrobials commonly used and factors that may lead to resistance spread in their community. Three veterinarians who had worked at the local animal clinic were asked to name the veterinary antimicrobials they had commonly prescribed for use in the area.

### 2.6 Sample collection

One hundred (*n* = 100) cattle belonging to the interviewed farmers were randomly selected for this study. These cattle were apparently healthy (i.e. no visible clinical signs) and at least 6 months old. The number of cattle to be sampled at each dip tank was determined using a proportional stratified sampling approach to ensure a good geographical spread of the sample. One to three cattle were randomly selected per farmer as the cattle passed through the race. A single rectal swab sample was obtained from each selected cattle using sterile dry culture swabs. On each day, samples were transported to the laboratory on ice within 3 hr of collection and then stored at −80°C for 1–3 weeks while awaiting processing.

### 2.7 Bacterial culture and isolation

The faecal swabs were inoculated onto Columbia agar with 5% horse blood and then onto MacConkey agar without crystal violet (Thermo Fischer Scientific, South Africa). The plates were then streaked out using the quadrant streaking method. The blood agar plates were incubated with 5% carbon dioxide and both plates were incubated at 37°C for 24 hr. Suspect *E. coli* and enterococci were subcultured on the blood agar and MacConkey agar without crystal violet to obtain pure cultures. Gram‐positive, catalase (Davies Diagnostics, South Africa) negative cocci occurring singly or in pairs and producing pinpoint red colonies on MacConkey (i.e. lactose fermenters) and positive for aesculin (Thermo Fischer Scientific, South Africa) hydrolysis were subjected to Streptococcal grouping using a commercial test kit, Streptex kit (Thermo Fischer Scientific, South Africa). Colonies falling in Lancefield group D were subjected to further sugar tests (Thermo Fischer Scientific, South Africa) to allow differentiation of some of the *Enterococcus* species according to the criteria described by Quinn, Carter, Markey, and Carter (1994). Some enterococci could not be identified to species level due to the limited array of sugars tested.

Gram‐negative, catalase‐positive and oxidase‐negative, medium‐sized rods producing large pink colonies on MacConkey agar were presumed to be *Enterobacteriaceae* if positive for indole (Merck, South Africa) production and then subjected to API 10S test (BioMerieux, South Africa) to identify *E. coli* isolates. One *E. coli* and/or one *Enterococcus* species if cultured were isolated from each animal sampled.

### 2.8 Antimicrobial susceptibility testing

A micro‐titre broth dilution method using water‐soluble antimicrobial powders (Sigma Aldrich, Germany) was used to determine the susceptibility profile of each isolate following the Clinical and Laboratory Standards Institute (CLSI) guidelines (CLSI, 2018a). *E. coli* isolates were subjected to antimicrobial susceptibility testing to the following agents; colistin sulphate, amoxicillin, enrofloxacin, chlortetracycline and gentamicin, while enterococci isolates were tested against amoxicillin, chlortetracycline, erythromycin, vancomycin and enrofloxacin (Sigma Aldrich, Germany). The tested antimicrobials fall under the group of antimicrobials recommended for routine antimicrobial surveillance testing studies for *E. coli* and enterococci (Caprioli et al., 2000 ; Franklin et al., 2001). Due to resource limitations, testing was limited to last resort antimicrobials (vancomycin and colistin), tetracycline commonly used by farmers in the study area and a few other antimicrobials. Water‐soluble antimicrobial powders were used. The weight of each antimicrobial powder to be used was calculated based on the percentage purity of the powder and then dissolved in sterile water. The antimicrobial stock solutions were further diluted with the Mueller Hinton broth as necessary. Each of the antimicrobials was tested in duplicate in serial twofold dilutions from 0.25 µg/ml to 32µg/ml. Clinical breakpoints were used to categorise isolates into susceptible, intermediate or resistant (Table 1) (CLSI, 2018b). *E. coli* ATCC #25922 and *E. faecalis* ATCC # 29,212 were used as controls. Isolates that were resistant to at least one antimicrobial were defined as resistant while those resistant to three or more antimicrobials were defined as multidrug resistant (Magiorakos et al., 2012).

**TABLE 1:** Minimum inhibitory concentration (MIC) breakpoints used for resistance classification of bacterial isolates (adapted from CLSI, 2018b)

	MIC Interpretive criteria (µg/ml)		
	Susceptible	Intermediate	Resistant
*E. coli*			
Amoxicillin^c^	≤8	16	≥32
Gentamicin	≤4	8	≥16
Tetracycline	≤4	8	≥16
Enrofloxacin^a^	≤0.25	0.5–1	≥2
Colistin^b^	≤2		≥4
Enterococci			
Amoxicillin^c^	≤8	—	≥16
Tetracycline	≤4	8	≥16
Erythromycin	≤0.5	1–4	≥ 8
Vancomycin	≤4	8–16	≥32
Enrofloxacin^a^	≤0.25	0.5–1	≥2

^a^ Breakpoint for bovine respiratory disease was used.

^b^ Colistin breakpoint for Pseudomonas aeruginosa was used.

^c^ Ampicillin breakpoint used.

### 2.9 Data analysis

The questionnaire responses and antimicrobial susceptibility profiles of the bacterial isolates were coded and entered into SPSS statistics 25 (IBM) for descriptive analysis, i.e. frequencies and proportions were computed for all variables and presented as graphs and tables.

## 3 RESULTS

### 3.1 Farmer questionnaire survey

#### 3.1.1 Demographic profile of interviewed farmers

In total, 70 farmers were interviewed with the majority of them being men (80%, 56/70). More than half (59%, 37/63) of the farmers were over 45 years and majority of them (91%, 50/55) indicated that they had been rearing cattle for more than 5 years. Besides cattle, some of the farmers indicated rearing poultry (61%, 43/70), goats (24%, 17/70) and pigs (4%, 3/70), but none (0%, 0/70) reared sheep.

#### 3.1.2 Knowledge on antimicrobials and antimicrobial use practices of farmers

Only one farmer (1%) indicated being aware of what an antimicrobial is but could not give an example of one. Among the listed antimicrobials, the farmers indicated using Terramycin^®^ (86%) and Hitet^®^ (43%), both of which are oxytetracyclines. The farmers sourced medication mainly from the local animal clinic (60%) and an agricultural retailer (34%) in a neighbouring town (Table 2).

**TABLE 2:** Knowledge on antimicrobials and antimicrobial use practices of interviewed farmers

	Number of respondents	Percentage of respondents
Do you know what an antimicrobial agent is	(*n* = 70)	
No	69	99
Yes	1	1
Could give an example of an antimicrobial	0	0
Antimicrobials farmers used in the last year	(*n* = 70)	
Terramycin^®^	60	86
Hitet^®^	30	43
Source of drugs	(*n* = 67)	
Corporative	23	34
Local animal clinic	40	60
Veterinarian	6	9
Animal health technicians	0	0
Villagers that sell drugs	0	0
Keep record of treatments given to their livestock	(*n* = 65)	
Yes	19	29
No	46	71
How long do you use antibiotics for	(*n* = 66)	
Until clinical signs stop	3	5
3 days	16	24
As indicated on the medicine use instructions	1	2
Until the drug is finished	46	70
Disposal of expired antimicrobials	(*n* = 62)	
Throw in the bin or garbage pit	20	32
Burn	1	2
Throw in the toilet	15	24
Return to place of purchase	17	27
Throw away (not specified where)	9	14
Do you know the importance of observing a withdrawal period	(*n* = 67)	
Yes	48	72
No	19	28
Reasons given for observing a withdrawal period	(*n* = 48)	
Because the drug may affect humans	30	63
To check if animal has fully recovered	15	31
To prevent contracting the disease	1	2
Simply because vets and AHTs instruct them to	2	4

Twenty‐nine per cent of the farmers kept treatment records for their cattle. Twenty‐seven per cent of farmers indicated that they return expired drugs to place of purchase, 2% burn them, while the rest throw them away (Table 2).

Seventy‐two per cent of the farmers indicated that they were aware of the importance of observing a withdrawal period after treating animals. Of these, 63% highlighted that it was important to do so because the drug will still be in the body of the animal and may affect them (Table 2).

Farmers were also asked if they had used drugs to treat for conditions such as diarrhoea, fever, coughing, mastitis, abscess or to prevent tick‐borne diseases in the last year. Seven farmers (10%, 7/70) in total indicated having treated for one of the listed conditions. The farmers indicated treating for diarrhoea (6%, 4/70), fever (1%, 1/70), coughing (1%, 1/70) and for tick‐borne disease prevention (1%). All seven farmers indicated that response to treatment was good. The farmers could not recall the drugs that they had used for treatment of the different ailments except for one farmer who indicated using Terramycin^®^ (an oxytetracycline). In South Africa, Terramycin^®^ is available as an over‐the‐counter medication without veterinary prescription. Therefore, efforts to identify if the farmers managed to buy prescription drugs without prescriptions as a follow‐up question were thus futile. It is possible that the administered drugs the farmers failed to recall may have not been antimicrobials.

#### 3.1.3 Knowledge on antimicrobial resistance (AMR)

Forty‐two per cent of the farmers had heard about antimicrobial resistance with their main source being veterinarians and AHTs (82%). Of these, only one farmer (4%, 1/28) indicated that antimicrobial resistance involves microorganisms becoming resistant to treatment. However, the same farmer indicated that the body of the animal becomes resistant to treatment; hence, none of the farmers have a clear understanding of what antimicrobial resistance involves (Table 3). All the farmers indicated interest in learning more on prudent use of antimicrobials.

**TABLE 3:** Knowledge on antimicrobial resistance (AMR) of interviewed farmers

	Number of respondents	Percentage of respondents
Have you heard about AMR	(*n* = 67)	
Yes	28	42
No	39	58
Source of information on AMR	(*n* = 28)	
Health workers (veterinarians and AHTs)	23	82
Television/radio	3	11
Farmer's day talk	2	7
Know any use practices that select for AMR development	(*n* = 28)	
	0	0
With AMR, the body becomes resistant to treatment	(*n* = 28)	
Yes	1	4
No	27	96
With AMR, microorganisms become resistant to treatment	(*n* = 28)	
Yes	1	4
No	27	96

#### 3.1.4 Animal health technicians and veterinarians’ interviews

Two AHTs were interviewed and both indicated that tetracyclines were the most commonly used antimicrobials in the community. Both rarely encountered cases of antimicrobial treatment failure but rated owner compliance with antimicrobial use instructions as poor. Both AHTs indicated underdosing by farmers as the main concern. One AHT also indicated the use of wrong routes of administration; inappropriate storage of drugs and use of expired drugs as areas of concern.

Three veterinarians (*n* = 3) contacted indicated that tetracyclines were commonly used while penicillins, sulphonamides and enrofloxacin were occasionally used.

### 3.2 Bacterial isolation and antimicrobial susceptibility

#### 3.2.1 Bacterial isolation

Seventy‐nine per cent (79/100) of the cattle were culture positive for *E. coli* while 71% (71/100) were culture positive for *Enterococcus* species. One *Escherichia coli* and/or one *Enterococcus* were isolated from each culture‐positive animal sample. A total of 150 bacterial isolates; 79 *Escherichia coli* and 71 enterococci were obtained. Forty‐one per cent (29/71) of the enterococci isolates were not identified to species level. Of the speciated enterococci, *Enterococcus* *faecium* (79%; 33/42) was dominant followed by *E. faecalis* (10%; 3/42), *E. durans* (10%; 3/42) and *E. avium* (10%; 3/42).

#### 3.2.2 Antimicrobial susceptibility of *Enterococcus* species

**FIGURE 1:**
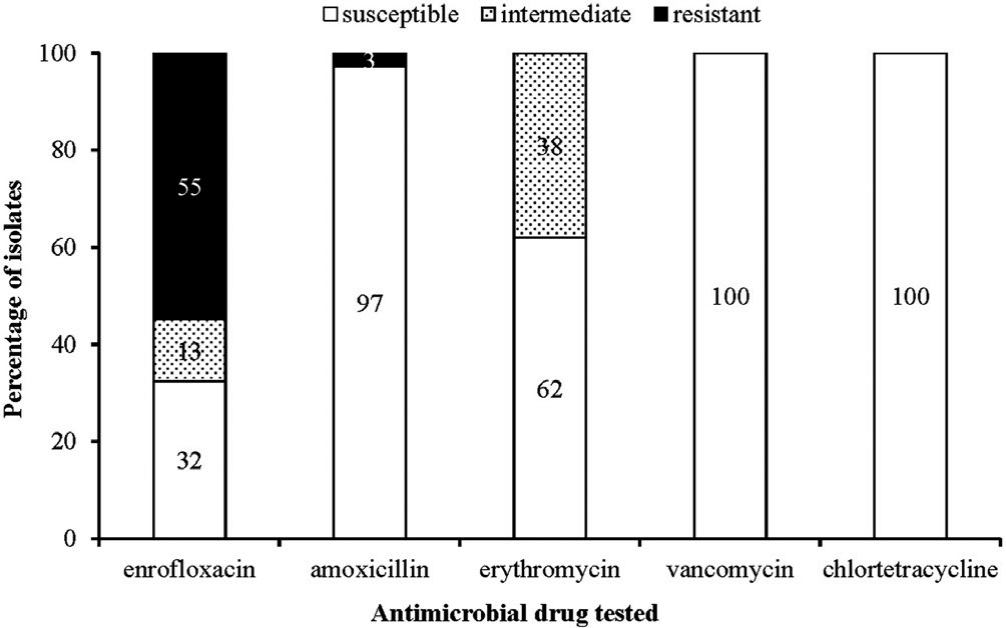
Antimicrobial susceptibility profile to five antimicrobials of *Enterococcus* isolates (*n* = 71) from healthy cattle

**FIGURE 2:**
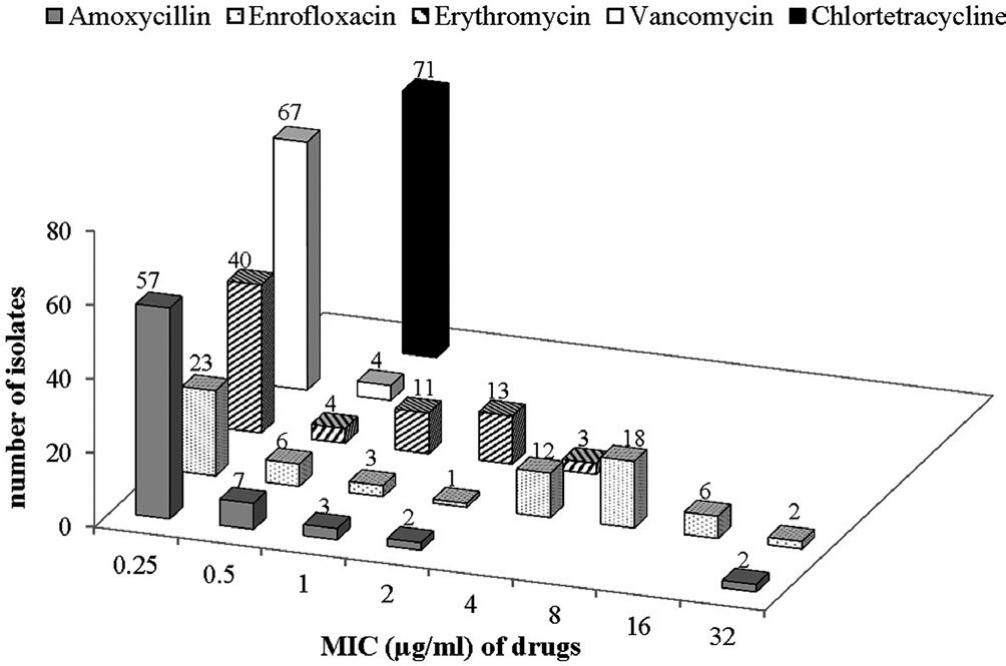
Minimum inhibitory concentrations of five antimicrobials for *Enterococcus* isolates (*n* = 71) from healthy cattle

Fifty‐five per cent (39/71) of the *Enterococcus* isolates were resistant to at least one antimicrobial with enrofloxacin resistance (55%; 39/71) dominating followed by amoxycillin resistance (3%; 2/71). All (100%, 71/71) the enterococci isolates were susceptible to chlortetracycline and vancomycin (Figure 1). Among the *Enterococcus* species, only *E. faecium* isolates (6%; 2/33) were resistant to amoxycillin while all species except for *E. faecalis* were resistant to enrofloxacin (Table 4). Among the antimicrobials tested against the enterococci isolates, vancomycin and chlortetracycline had very narrow MIC distributions. Enrofloxacin had the widest MIC range which had a bimodal distribution with a cluster of susceptible/intermediate strains and a cluster of resistant strains (Figure 2). Two resistance patterns were observed among the enterococci isolates; resistance to enrofloxacin only in (52%; 37/71) and enrofloxacin‐amoxycillin resistance in 3% (2/71) of the isolates. No multidrug resistant strains were detected.

**TABLE 4:** Antimicrobial susceptibility profile of *Enterococcus*species (*n* = 71) from healthy cattle

Species	∑*n*	Amoxycillin *n* (%)			Enrofloxacin *n* (%)			Chlortetracycline *n* (%)			Vancomycin *n* (%)			Erythromycin *n* (%)		
		S	I	R	S	I	R	S	I	R	S	I	R	S	I	R
*E. faecium*	33	31 (94)	0	2 (6)	2 (6)	1 (3)	30 (91)	33 (100)	0	0	33 (100)	0	0	12 (36)	21 (64)	0
*E. faecalis*	3	3 (100)	0	0	2 (67)	1 (33)	0	3 (100)	0	0	3 (100)	0	0	2 (67)	1 (33)	0
*Enterococcus* spp.	29	29 (100)	0	0	18 (62)	8 (28)	3 (10)	29 (100)	0	0	29 (100)	0	0	27 (93)	2 (7)	0
*E. avium*	3	3 (100)	0	0	1 (33)	0	2 (67)	3 (100)	0	0	3 (100)	0	0	1 (33)	2 (67)	0
*E. durans*	3	3 (100)	0	0	0	0	3 (100)	3 (100)	0	0	3 (100)	0	0	2 (67)	1 (33)	0

Note

∑*n* = Total number of isolates tested.

*n* = Number of isolates tested.

(%)^Ϫ^ = Percentage of isolates.

S, susceptible; I, intermediate; R, resistant, *n*, number of isolates.

#### 3.2.3 Antimicrobial susceptibility of *E. coli* isolates

**FIGURE 3:**
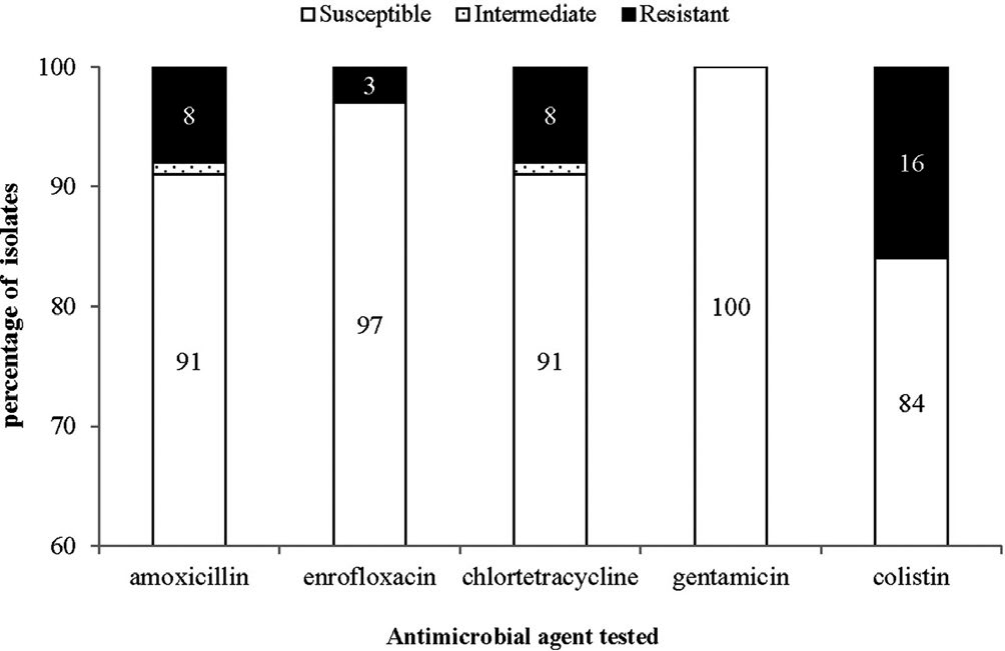
Antimicrobial susceptibility to five antimicrobials of *E. coli* isolates from healthy cattle

**FIGURE 4:**
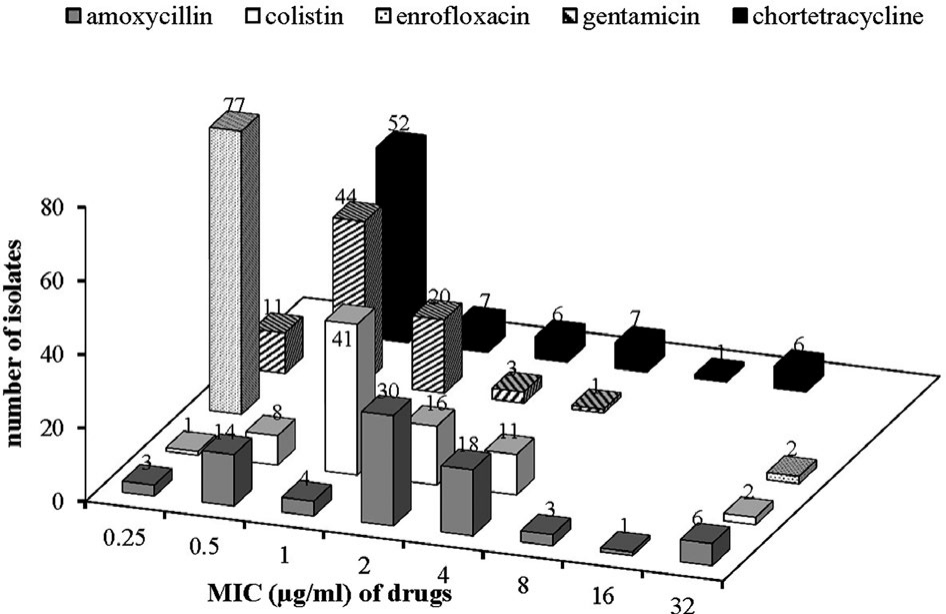
Minimum inhibitory concentrations of five antimicrobials for *E. coli* (*n* = 79) isolated from healthy cattle

Twenty‐seven per cent (21/79) of the *E. coli* isolates were resistant to at least one antimicrobial. The highest level of resistance detected was against colistin (16%; 13/79). All (100%; 79/79) of the isolates were susceptible to gentamicin (Figure 3). All the antimicrobials tested against the *E. coli* isolates had a wide MIC distribution except for enrofloxacin (Figure 4). Seven phenotypic resistance patterns were detected in the *E. coli* isolates (Table 5). Amoxycillin‐chlortetracycline resistance was the dominant co‐resistance phenotype. No multidrug resistant strains were detected.

**TABLE 5:** Phenotypic antimicrobial resistance patterns of *E. coli* isolates (*n* = 79) from healthy cattle

Resistance pattern	Number of isolates	Percentage of isolates
Amoxycillin	1	1%
Enrofloxacin	1	1%
Chlortetracycline	1	1%
Colistin	11	14%
Chlortetracycline–colistin	1	1%
Chlortetracycline–amoxycillin	4	5%
Enrofloxacin–colistin	1	1%

The minimum inhibitory concentrations of the antimicrobials for the ATCC control strains fell within the CLSI ranges.

## 4 DISCUSSION

### 4.1 Questionnaire surveys

Containing antimicrobial resistance requires the input of everyone in society (World Health Organisation, 2015b) with farmers being among the individuals with a significant role to play. In this study, only one farmer (1%) knew what an antimicrobial is, despite many of them using antimicrobials. This ignorance is concerning as farmers might not take the necessary precautions when using antimicrobials or may use them injudiciously. A clear understanding of how antimicrobials function and when their use is indicated is a prerequisite for judicious use behaviour (Ancillotti et al., 2018). Failure of the farmers in this study to describe antimicrobials may be partly due to lack of a vernacular term denoting them (Chauhan et al., 2018). Therefore, introduction of vernacular terms that delineate antimicrobials may be helpful.

Tetracyclines, sulphonamides, cloxacillin intramammary, fosfomycin, tylosin and kitasamycin are available as over‐the‐counter drugs in South Africa, (Eagar, Swan, & Van Vuuren, 2012; Naidoo, 2009). Similar to our study, tetracyclines were reportedly the most commonly used antimicrobials by small‐scale farmers in several African countries including South Africa (Eagar et al., 2012; Moneoang & Bezuidenhout, 2009); Zambia (Mainda et al., 2015; Mubita et al., 2008) and Tanzania (Caudell et al., 2017; Katakweba, Mtambo, Olsen, & Muhairwa, 2012; Nonga, Mariki, Karimuribo, & Mdegela, 2009). The popularity of tetracyclines among livestock farmers is not surprising because they are relatively inexpensive and readily available without prescription in South Africa and are used for the treatment of various clinical disease conditions (Henton et al., 2011; Moneoang & Bezuidenhout, 2009) including endemic tick‐borne diseases such as anaplasmosis and heartwater (Aubry & Geale, 2011; Yunker, 1996).

Despite tetracyclines being the popular antimicrobial used by the farmers, we observed low levels of resistance to tetracyclines. Other investigators have made similar findings among small‐scale famers (Mainda et al., 2015; Mubita et al., 2008). One of the limitations of this study was that we did not investigate the volumes and frequency of antimicrobial use. Perhaps the use of the tetracyclines by the farmers in our study was not frequent enough to exert adequate selective pressure for resistance development.

All the farmers in this study sourced their veterinary drugs from legally established distributors. This is a positive finding since antimicrobials sold by legal distributors in South Africa are expected to be registered with the relevant authorities and are therefore quality assured (Eagar et al., 2012). This significantly reduces the risk of resistance development due to use of poor‐quality agents. Small‐scale dairy farmers in Zambia (91%) and rural Peru (87.8%) sourced their drugs from veterinarians and veterinary drug stores (Mainda et al., 2015; Redding et al., 2014). Adequate training of veterinary drug salesmen on animal health and disease management, and prudent drug use is thus critical since these people may at times be the only animal health informants at the farmers’ disposal.

A clear understanding of antimicrobial resistance and its consequences in animals and humans is a significant motivator for prudent use of antimicrobials (Eltayb, Barakat, Marrone, Shaddad, & Stålsby Lundborg, 2012; Sirdar, Picard, Bisschop, & Gummow, 2012). Therefore, it is worrying that half of the farmers were not aware of the public health importance of observing withdrawal periods. In addition, some of the withdrawal periods observed by the farmers fell short of the recommended periods for the tetracyclines they indicated using.

Among the farmers (70%) who indicated using antimicrobials until the bottle is empty, it is possible that some of them were referring to use of an antimicrobial or even other veterinary agents in general as opposed to duration of antimicrobial use when treating a sick animal. Nonetheless, poor compliance with antimicrobial use instructions, poor record keeping of treated animals and improper disposal of expired antimicrobial drugs by farmers in this study are likely to increase the development of antimicrobial resistance (Bound & Voulvoulis, 2005). The use of incorrect doses and routes of administration is also likely to increase the risk of the development of resistance and exposure of the public to antimicrobial residues (Khatun et al., 2018). Farmers must be encouraged to keep good records as they are an important means of monitoring both disease burden and antimicrobial use on the farm (Speksnijder, Mevius, Bruschke, & Wagenaar, 2015). Safe disposal of antimicrobials is also imperative to avoid environmental contamination and increased antimicrobial resistance selection pressure (Bound & Voulvoulis, 2005).

Although some farmers indicated hearing about antimicrobial resistance, they may have been referring to drug resistance in general given that the farmers indicated not being aware what an antimicrobial is. Antimicrobial resistance awareness campaigns should be developed with the help of veterinarians and AHTs working in the area as farmers recognise them as their main source of information on antimicrobial use.

### 4.2 Antimicrobial susceptibility of bacterial isolates

#### 4.2.1 *Enterococcus* species

*Enterococcus faecium* was the most common *Enterococcus* species isolated in this study. Similar finding has been reported in cattle in Nigeria (Ngbede, Raji, Kwanashie, Kwada, & Kwaga, 2017). In contrast, *E. hirae* was the most common species in dairy cattle in a previous study in South Africa (Tanih, 2016). The differences between studies may be related to diet and variation in the environmental microbiomes to which the different cattle populations are exposed (Anderson, Parrish, Akhtar, Zurek, & Hirt, 2008) as well as use of different culture media (Jackson, Fedorka‐Cray, Jackson‐Hall, & Hiott, 2005). Polymerase chain reaction would have been helpful in identifying the non‐speciated enterococci to species level.

Low levels of resistance to the tested antimicrobials were detected among the enterococci with the exception of enrofloxacin. Similarly, high resistance to enrofloxacin (90%) was observed in cattle in the South African National Veterinary Surveillance and Monitoring Program (SANVAD) (van Vuuren et al., 2007). In contrast, higher levels of resistance to tetracycline (26.7%–100%), erythromycin (20%–88.9%) and vancomycin (20%–33%) were detected in South Africa at selected commercial dairy farms (Tanih, 2016) and in the SANVAD. In addition, resistance to ampicillin (40% for *E. faecium*) was high in the SANVAD (van Vurren et al., 2007).

#### 4.2.2 Escherichia coli

The proportion of faecal samples from which *E. coli* was isolated in this study was lower than expected considering that it is one of the dominant commensal flora of the gastrointestinal tract of cattle. This could be due to the drying of swabs due to lack of transport medium (Centre for Disease Control & Prevention, 1994).

The level of resistance to tetracycline, amoxycillin, enrofloxacin and gentamicin in *E. coli* isolates in this study was generally low. Similarly, low levels of resistance (0.8%–4.2%) to these antimicrobials were detected in the SANVAD with the exception of tetracycline resistance which was higher (33.6%) (van Vuuren et al., 2007). Our results were also similar to the low levels of resistance reported in cattle in Zambia (Mainda et al., 2015; Mubita et al., 2008).

The level of colistin resistance detected in the *E. coli* isolates was unexpected considering that the study focused on a rural communal farming area. However, colistin resistance has previously been detected in *E. coli* isolates from hosts not previously exposed to colistin or any antimicrobial treatment (Bachiri et al., 2018; Liakopoulos, Mevius, Olsen, & Bonnedahl, 2016; Ruzauskas & Vaskeviciute, 2016).

Colistin has a tendency to adsorb to laboratory plastic ware resulting in reduced availability of the antimicrobial in assays (Humphries, 2015; Karvanen, Malmberg, Lagerbäck, Friberg, & Cars, 2017). This might have falsely elevated minimum inhibitory concentrations (MICs) resulting in overestimation of resistance levels in this study. Furthermore, lack of an intermediate susceptibility category for colistin might have also led to false resistant categorisation of isolates with minimum inhibitory concentrations close to the resistance breakpoint (Matuschek, Åhman, Webster, & Kahlmeter, 2018). Nonetheless, the high level of colistin resistance observed in this study warrants further molecular investigation.

The most common co‐resistance phenotype in the *E. coli* isolates in this study was amoxycillin–chlortetracycline resistance as was the case in the study in dairy cattle in Zambia (Mainda et al., 2015). This co‐resistance may be due to resistance genes being borne on the same mobile genetic elements and these can disseminate rapidly resulting in increased resistance levels.

### 4.3 Study limitations

Despite the important findings from the questionnaire survey, it has some shortcomings. The responses in this questionnaire survey were self‐reported and thus may be subject to recall bias (Caudell et al., 2017). Ignorance of what antimicrobials are potentially biased some of the responses in that perhaps the farmers were in some instances making reference to drugs in general as opposed to antimicrobials specifically. The small cattle sample size may have influenced the resistance levels detected in this study.

## 5 CONCLUSION

The level of antimicrobial resistance in this study was generally low. Of concern is the level of colistin‐resistant *E. coli* isolates detected. Further molecular investigation is warranted to check if the detected colistin resistance is plasmid mediated. Over‐the‐counter availability of antimicrobials must be accompanied by tailor‐made farmer education programmes that raise awareness on prudent antimicrobial use and antimicrobial resistance to help promote responsible antimicrobial use among farmers. However, it is important to note that this awareness alone does not guarantee behavioural change. These awareness campaigns should be supported by other interventions including primary animal health training programmes for farmers and policy changes.

## CONFLICT OF INTEREST

The authors have no conflicts of interest to report.

## AUTHOR CONTRIBUTION

**Charlotte Ropafadzo Mupfunya:** Investigation; Methodology; Writing‐original draft; Writing‐review & editing. **Daniel Nenene Qekwana:** Methodology; Supervision; Writing‐review & editing. **Vinny Naidoo:** Conceptualization; Funding acquisition; Methodology; Supervision; Writing‐review & editing.

6

### 6.1 PEER REVIEW

The peer review history for this article is available at https://publons.com/publon/10.1002/vms3.334.

## Supporting information

Data S1
