# Supplementary material for: Antimicrobial use practices and resistance in indicator bacteria in communal cattle in the Mnisi community, Mpumalanga, South Africa
Source: Vet Med Sci. 2020 Aug 31;7(1):112–21. doi: 10.1002/vms3.334 (PMC7840202; doi:10.1002/vms3.334)
Supplement: Supplementary file 1 — Data S1 [file VMS3-7-112-s001.docx]

# Antimicrobial Resistance in Small Scale Farms

**Farm questionnaire**

**Purpose of survey**

The purpose of this survey is to assess how antimicrobials are used in your region. The answers you provide will be helpful in identifying the areas where we may be of assistance. Your personal details will be kept strictly confidential and we will prepare a report from the responses provided. Your participation in this survey is greatly appreciated.

# Instructions

For questions with ready-made answers, please tick the relevant answer(s).

Date………………………………………………………….. Stock card number……………………………………………

**Sex** female
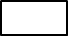
 male

# Age group

18 – 25 years
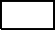
26 – 35 years
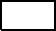
 36 – 45 years
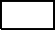
over 46 years
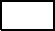


1. State the number of years that production animals have been kept on your household.

………………………………………………………………………………………………

1. **a)** What production animal species are currently kept on the household?

Cattle Goats Sheep Pigs Poultry

- 1. How many cattle are kept on your household?

…………………………………………………………………………………………………..

- 1. Where did you source additions to your cattle herd if any were made in the last year?

Within the same village
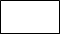
 Other villages in study area
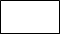
 Outside study area
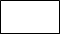
 No additions were made
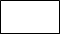


1. **a)** Do you know what an antimicrobial drug is? **Yes
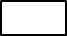
 No
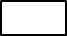
**
2. **If your response to 3.a) is yes**, **list three** (3) antimicrobial drugs that you know 1………………………………………………………. 2………………………………………………………. 3……………………………………………………….
3. Tick the drugs you have used on your animals in the last year;
4. Terramycin
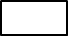

5. Depocillin
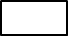

6. Depomycin
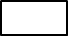

7. Oxytetracycline
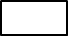

8. Hitet
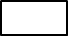

9. Sulfatrim
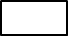

10. Trivetrin
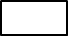

11. Intertrim
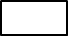

12. Dofatrim
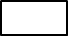

13. Where do you source the drugs you use on your animals? *(tick all the relevant answers)*

Pharmacy/drug store Local animal clinic Mobile salesman Veterinary doctor

Animal Health Technician

**5.a** If you have treated any of your cattle for one of the following conditions in the table below **in the last year**, which drugs did you use and what were the routes of administration and the outcomes of the treatments?

| **Condition/indication** | **Drug(s) used** | **Route of administration** | **Outcome of the treatment** | | |
| --- | --- | --- | --- | --- | --- |
|  |  |  | **Animals recovered** | **Animals still sick** | **Animals died** |
| cough |  |  |  |  |  |
| High fever |  |  |  |  |  |
| mastitis |  |  |  |  |  |
| Swelling or abscess |  |  |  |  |  |
| diarrhoea |  |  |  |  |  |
| To prevent infection |  |  |  |  |  |
| To prevent tickborne diseases |  |  |  |  |  |

**b.** Of the drugs in **5.a,** list those that required a prescription for purchase.

………………………………………………………………………………………………………

………………………………………………………………………………………………………

………………………………………………………………………………………………………

………………………………………………………………………………………………………

………………………………………………………………………………………………………

……………………………………………………………………………………………………..

**6.** Do you keep a record of the treatments you give to your animals

# Yes
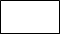
 No
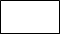


1. How long do you wait from the last treatment to slaughtering the animals for food

………………………………………………………………………………………………..

1. Do you know the importance of waiting for a while from the last treatment to slaughtering animals for food or drinking milk from the animals? **Yes
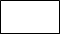
 No
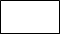
**
2. **If your response to 9** is yes, give one reason why it is important to wait from the last treatment to slaughtering the animals for food.

………………………………………………………………………………………………………

………………………………………………………………………………………………………

………………………………………………………………………………………………………

1. How long do you usually use the antibiotics for?

until clinical signs stop
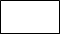
 up to the end of the indicated antibiotic treatment course on the box or label
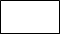
 For three days
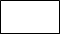


Until the bottle is empty
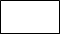


1. How do you dispose of expired antibiotics?

………………………………………………………………………………………………………

1. Have you heard/read about antimicrobial resistance?

# No
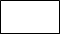


If **Yes,** tick all the relevant answers below

From health workers
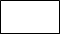
 On tv/radio
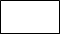
 From a pamphlet or newspaper or magazine
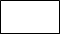
 From farmers’ day
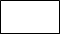
 From veterinary students who come to the farm
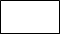


1. If you have heard/read about antimicrobial resistance, answer the following questions,

a) Antimicrobial resistance involves;

1. Infections becoming resistant to treatment by antimicrobial drugs. Yes
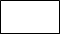
 No
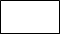

2. The body of the animal becoming resistant to antimicrobial drugs. Yes
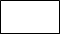
 No
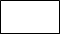


**b)** State any two antibiotic use practices that promote antimicrobial resistance development.

………………………………………………………………………………………………………

………………………………………………………………………………………………………

………………………………………………………………………………………………………

1. Are you interested in short courses on safe use of antimicrobial agents?

# Yes
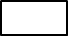
 No
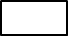


**AHT questionnaire**

**Purpose of survey**

The purpose of this survey is to assess how antimicrobials are used in your region. The answers you provide will be helpful in identifying the areas where we may be of assistance. Our aim is to help put together a treatment guide, that will make treatment of animals in your area easier. Your personal details will be kept strictly confidential and we will prepare a report from the responses provided. Your participation in this survey is greatly appreciated.

**Instructions**

For questions with ready-made answers, please tick the relevant answer(s).

Date…………………………………………………………..

**Sex** female
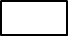
 male

**Age group**

18 – 25 years 26 – 35 years 36 – 45 years over 46 years

**1.**State the number of years that you have been serving in Study area as an AHT.

………………………………………………………………………………………………

**2.**Name **six** antibiotics you use the most to treat animals in Study area. ……………………………………………………………………………………………………… ………………………………………………………………………………………………………………………………………………………………………………………………………………………………………………………………………………………………………………………………………………………………………………………………………………………………

**3.** Do you encounter cases of antimicrobial treatment failure?

Never **
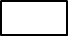
** Sometimes **
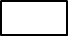
**

Often **
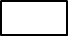
** Rarely **
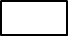
**

**4.** How often do you attend to cases of owner initiated antimicrobial treatment?

Never **
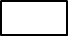
** Sometimes **
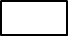
**

Often **
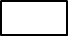
** Rarely **
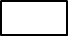
**

**5.** How do you rate owner compliance to recommended antimicrobial use practices?

Poor **
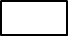
** satisfactory **
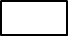
** good **
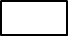
**

**6.** Are you interested in short courses on safe use of antimicrobial agents? **Yes
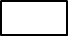
 No
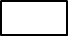
**

**7.** Highlight factors that you have identified that can lead to spread of antimicrobial resistance in Study area.

………………………………………………………………………………………………………………………………………………………………………………………………………………………………………………………………………………………………………………………………………………………………………………………………………………………………………………………………………………………………………………………………………
